# Neurotrophins as Therapeutic Agents for Parkinson’s Disease; New Chances From Focused Ultrasound?

**DOI:** 10.3389/fnins.2022.846681

**Published:** 2022-03-25

**Authors:** Alessandro Stefani, Mariangela Pierantozzi, Silvia Cardarelli, Lucrezia Stefani, Rocco Cerroni, Matteo Conti, Elena Garasto, Nicola B. Mercuri, Carmine Marini, Patrizia Sucapane

**Affiliations:** ^1^Department of System Medicine, Parkinson Center, University Tor Vergata, Rome, Italy; ^2^Department of System Medicine, UOC Neurology, University Tor Vergata, Rome, Italy; ^3^UOC Neurology and Stroke Unit, University of L’Aquila, L’Aquila, Italy; ^4^Parkinson’s and Movement Disorder Center, L’Aquila, Italy

**Keywords:** neurotrophins, movement disorders, neurodegeneration, MRgFUS, BBB

## Abstract

Magnetic Resonance–guided Focused Ultrasound (MRgFUS) represents an effective micro-lesioning approach to target pharmaco-resistant tremor, mostly in patients afflicted by essential tremor (ET) and/or Parkinson’s disease (PD). So far, experimental protocols are verifying the clinical extension to other facets of the movement disorder galaxy (i.e., internal pallidus for disabling dyskinesias). Aside from those neurosurgical options, one of the most intriguing opportunities of this technique relies on its capability to remedy the impermeability of blood–brain barrier (BBB). Temporary BBB opening through low-intensity focused ultrasound turned out to be safe and feasible in patients with PD, Alzheimer’s disease, and amyotrophic lateral sclerosis. As a mere consequence of the procedures, some groups described even reversible but significant mild cognitive amelioration, up to hippocampal neurogenesis partially associated to the increased of endogenous brain-derived neurotrophic factor (BDNF). A further development elevates MRgFUS to the status of therapeutic tool for drug delivery of putative neurorestorative therapies. Since 2012, FUS-assisted intravenous administration of BDNF or neurturin allowed hippocampal or striatal delivery. Experimental studies emphasized synergistic modalities. In a rodent model for Huntington’s disease, engineered liposomes can carry glial cell line–derived neurotrophic factor (GDNF) plasmid DNA (GDNFp) to form a GDNFp-liposome (GDNFp-LPs) complex through pulsed FUS exposures with microbubbles; in a subacute MPTP-PD model, the combination of intravenous administration of neurotrophic factors (either through protein or gene delivery) plus FUS did curb nigrostriatal degeneration. Here, we explore these arguments, focusing on the current, translational application of neurotrophins in neurodegenerative diseases.

## Introduction

Parkinson’s disease (PD) is a neurodegenerative disorder of aging, caused by the depletion of dopaminergic neurons in the substantia nigra pars compacta with consequent dopamine (DA) deficiency. The pathological trademark of PD is the eosinophilic cytoplasmic neuronal inclusions termed Lewy bodies, whose main component is abnormally folded α-synuclein (α-syn) protein aggregations (Wakabayashi et al., 2007). However, recent uncertainties and failures of protocols based on anti–α-syn antibodies (NCT03100149, NCT03318523, Antonini et al., 2020) reinforced the skepticism of those who rather emphasize the centrality of energy failure as the primum movens of disease pathogenesis; hence, the opportunity once more to validate neurorescue-based therapy including neurotrophins. Significantly, our group is actively challenging the metabolic production/accumulation of lactate as reliable biomarker for the progression of different neurodegenerative conditions (Liguori et al., 2015, 2016).

Consensus prevails on some seminal concepts:

- It is believed that PD pathology is a consequence of the interaction between genetic susceptibility and toxic environmental factors, but the factors responsible for the initiation of the pathophysiological cascade remain still largely unknown (for a review Henderson et al., 2019).

- In this scenario, no effective disease-modifying therapy is presently available for neurodegenerative disease such as PD and related sinucleinopaties. Current treatments for PD are symptomatic and, although effective in the management of motor symptoms, they do not counteract the progression of neurodegeneration and disease time-course; moreover, complications such as motor fluctuations dyskinesias are unescapable after years of replacing DA therapies (LeWitt, 2015).

- Albeit we are handling complex therapies as routine, and public sensibility of stakeholders drive new multidisciplinary approaches (Bloem et al., 2021), the unmet needs maintain their severe burden.

That said, the increasing understanding of PD pathophysiology led to refuel at considering the potential role of several molecules with neuroprotective and neurorestorative properties as rescue therapy, together with questioning on solutions, which may overcome the pitfalls encountered in previous trials.

Over the last decades rigorous and extensive research has tried to devise disease-modifying therapies for neurodegenerative extrapyramidal diseases. In this area, neurotrophic factors (NTFs), due to their action on promoting neuronal survival and neurite outgrowth, emerged as compounds potentially able to limit, or even stop, cell degeneration, improving functionality of stressed neurons (Chmielarz and Saarma, 2020). In fact, there is a converging view that insufficient neuronal reserve of NTFs may be, in part, involved in synaptic plasticity deficit and neurodegenerative diseases onset (Baquet et al., 2005; Zuccato and Cattaneo, 2009).

This brief review will examine the evidence collected in experimental models; hence, we will discuss the current limitations detected when trying to transfer those results in meaningful clinical success, finally questioning on the potential utilization of low-intensity focused ultrasound (LIFU) as non-routine key approach for NFTs delivery.

## A Brief Excursus Around Neurotrophins in Experimental Models of Parkinson’s Disease

NTFs are important regulators of neural survival, development, function, and plasticity. NTFs are classified into three main groups: (i) the neurotrophins, including the nerve growth factor (NGF), brain-derived neurotrophic factor (BDNF), neurotrophin-3 (NT-3), and neurotrophin-4 (NT-4); (ii) the glial cell line–derived neurotrophic factor (GDNF) family of ligands, including GDNF, neurturin (NRTN), artemin (ARTN), and persephin (PSPN); (iii) the neurokines, including ciliary neurotrophic factor (CNTF), interleukin-6 (IL-6), and cardiotrophin (CT-1) (Bothwell, 2014).

Given the fundamental role played by neurotrophins in regulating neuronal functions and their distribution in critical neuronal areas, it is not surprising that a significant number of psychiatric and neurodegenerative disorders is associated with altered NTFs’ levels and with changes in the expression of their receptors.

The interest in NTFs applied to PD dates back to the 1990s. Early studies in rat mesencephalic cultures found that BDNF prevents death of dopaminergic neurons, supporting their survival in the ventral tegmental area and medial substantia nigra (SN), also promoting their differentiation into dopaminergic cells (Hyman et al., 1991; Beck et al., 1993; Murer et al., 1999; Baquet et al., 2005). Besides the well-established neurotrophic action, BDNF also possesses other neuroprotective activities, such as anti-apoptosis, anti-oxidation, suppression of autophagy, and restoration of mitochondrial dysfunction (Chen et al., 2017; Miller et al., 2021).

Multiple lines of pre-clinical evidence documented abnormal expression of BDNF in different PD animal models proving that neurotrophins become downregulated, although the results of different studies were not always in complete agreement, probably for the different experimental settings (Collier et al., 2005; Mocchetti et al., 2007; Berghauzen-Maciejewska et al., 2015; Sampaio et al., 2017).

Silencing the gene encoding BDNF in mice resulted in the loss of dopaminergic neurons and in motor impairments, which confirms the role of BDNF in the deterioration of motor and cognitive abilities in PD protecting neurons against degeneration (Baker et al., 2005; Baquet et al., 2005). However, controversial results on neuronal dopaminergic protection and recovery of DA levels were obtained from different animal models of PD (Levivier et al., 1995; Lucidi-Phillipi et al., 1995; Galpern et al., 1996; Klein et al., 1999). A fairly recent study by Hernandez-Chan et al. (2015) used a delivery method called neurotensin-polyplex that utilized the neurotensin receptors for the internalization of nano vesicles specifically in dopaminergic neurons. The delivery of BDNF after the 6-hydroxydopamine (6-OHDA)–induced lesion showed substantial improvement in parkinsonian behavior related to a recovery of striatal DA. Moreover, the significant sprouting of dopaminergic fibers and the absence of recovery of DA level and number of surviving tyrosine hydroxylase (TH)–positive cells in the SN support the conclusion that BDNF was unable to stimulate neurogenesis but can sustain neuritogenesis both in the SN and striatum, in line with previous studies (Klein et al., 1999; Somoza et al., 2010).

Apart from the studies focused on BDNF expression in PD, also, the role of other NTFs has been widely investigated including the GDNF and its family member NRTN.

Different from BDNF, whose distribution is diffused in frontal cortex, hippocampus, basal ganglia, cerebellum, SN, and other brainstem regions (He et al., 2013), GDNF is only expressed in the dorsal and ventral striatum, anteroventral nucleus of the thalamus, septum, and subcommissural organ (Pascual et al., 2011). Curiously, there are no GDNF receptors mRNAs in the striatum, but they are highly expressed in the nigral cells (Trupp et al., 1997), suggesting a specific action on nigral dopaminergic neurons.

GDNF resulted to be more potent than BDNF on survival of SN pars compacta (SNpc) dopaminergic neurons in the brain of lesioned animal models of PD (Lu and Hagg, 1997; Rosenblad et al., 2000; Sun et al., 2005); GDNF is also able to stimulate axonal sprouting of lesioned SNpc neurons, but less effective in promoting striatal reinnervation or functional recovery in 6-OHDA lesion model (Rosenblad et al., 2000).

The therapeutic benefits of this NTF have been demonstrated to be effective in different neurotoxin-induced [6-OHDA and 1-methyl-4-phenyl-1,2,3,6-tetrahydropyridine (MPTP)] models of PD both in rodents (Choi-Lundberg et al., 1997; Tenenbaum and Humbert-Claude, 2017) and in non-human primates (Gash et al., 1996). In addition, the GDNF-family ligand NRTN has a neuroprotective effect, similar to GDNF, both on damaged nigral dopaminergic neurons in an animal model of PD and on ventral mesencephalic dopaminergic neurons *in vitro* (Horger et al., 1998; Fjord-Larsen et al., 2005). Nevertheless, NRTN diffuses less efficiently than GDNF in the parenchyma, due to its higher heparin-binding properties (Hadaczek et al., 2010; Runeberg-Roos et al., 2016). Recently, NRTN variants with lower affinity to heparin were showed to have increased brain biodistribution, to have increased chemical stability, and to be more efficient than GDNF in 6-OHDA rat model (Runeberg-Roos et al., 2016).

The inability of NTFs to cross the blood–brain barrier (BBB), the poor diffusion into brain tissues due to their large molecular size, together with a short half-life, led to the development of different strategies to improve the delivery of NTFs in a cell-specific and inducible manner, including cell grafts and viral vectors.

The implantation of genetically engineered human fibroblasts, protected by a semipermeable polymer capsule, enabled the direct production of GDNF in the striatum of a bilateral 6-OHDA lesion rat model, resulting in a significant improvement of movement performance associated with striatal reinnervation of TH-positive fibers (Sajadi et al., 2006). Recently, a novel strategy used genetically modified hematopoietic stem cell transplantation, capitalizing the propensity of derived macrophages to home to sites of neurodegeneration: macrophage-mediated GDNF delivery protected against dopaminergic neurodegeneration and lead to significant reversal of both motor and non-motor dysfunction in non-neurotoxin mice model of PD (Chen C. et al., 2019).

Delivery *via* viral vectors has the clear advantage that the expression can be elicited only in selected cells, which might mimic a more natural distribution of the ligand inside the tissue. In the last decades, different kinds of virus vectors were engineered to sustained long-term expression of NTFs in the treatment of neurodegenerative diseases (Kordower and Bjorklund, 2013). The viral strategies have been improved to obtain a temporal and quantity control of GDNF expression with the aim to avoid compensatory mechanisms on DA homeostasis, such as downregulation of TH transcription (Georgievska et al., 2002). Neuroprotective effects in the absence of TH downregulation have been obtained by applying low-GDNF doses either by injecting a low amount of viral vector (Eslamboli et al., 2005) or by controlling the level of transgene expression through a inducible viral vector (Tereshchenko et al., 2014; Chtarto et al., 2016).

More recently, cerebral dopamine neurotrophic factor (CDNF) and mesencephalic astrocyte-derived neurotrophic factor (MANF) formed a new, evolutionarily conserved family of NTFs due to their unique structures and potent protection of embryonic DA neurons (Parkash et al., 2009). In mammals, CDNF expression occurs broadly in the central nervous system and in peripheral tissues, including the cerebral cortex, hippocampus, cerebellum, thalamus, SN, and striatum, showing a partial overlap with MANF expression (Lindholm et al., 2007, 2008). Different from other NTFs, CDNF and MANF are partially retained in the lumen of endoplasmic reticulum (ER) and their primary function appears to be the modulation of the unfolded protein response (UPR) pathway that is activated in response to ER stress (Jäntti and Harvey, 2020). Prolonged ER stress is associated with the onset and progression of chronic neurodegenerative diseases, including PD and Alzheimer’s disease.

CDNF and MANF diffuse slightly better than GDNF in the brain tissue (Voutilainen et al., 2011) and protect nigrostriatal DA neurons from 6-OHDA–sinduced degeneration if injected in the striatum both before and after the induction of the lesion (Lindholm et al., 2007; Voutilainen et al., 2009). However, data from severe rat 6-OHDA neurorestoration models of PD suggest that CDNF could be more efficient than MANF (Voutilainen et al., 2011).

The neuroprotective and the neurorestorative effects of CDNF have also be demonstrated in the mouse MPTP model of PD (Airavaara et al., 2012) and through gene therapy strategies using adenovirus-associated virus (Bäck et al., 2013; Ren et al., 2013). However, contrary to previous results, the intrastriatal delivery of CDNF and MANF by lentiviral vector showed no beneficial effect on 6-OHDA rat model of PD; only the combined intranigral viral delivery of both factors was able to improve motor deficit and to lead to greater increase in striatal TH-positive fiber and significant protection of DA neurons in the SN (Cordero-Llana et al., 2015).

In addition to neurotrophic effects, recent studies on primary glial cell cultures and in 6-OHDA rat model of PD suggest that CDNF has an anti-inflammatory effect alleviating ER stress and reducing nitrosative stress and pro-inflammatory cytokines (Cheng et al., 2013; Nadella et al., 2014).

Frequently, pre-clinical studies on NTFs showed controversial results mainly due to different experimental settings and animal models. The dose of NTFs and the duration of the administration are important variables to take into account, and it was one of the main pitfalls for GDNF. Indeed, the sustained striatal overexpression of GDNF in lesioned animal has been associated with aberrant sprouting of the regenerating dopaminergic fibers and reduced DA synthesis, resulting in undesirable behavioral effects (Georgievska et al., 2002; Sajadi et al., 2005). Similarly, also, the long-term effects of elevated BDNF levels should be considered: an earlier study documented hypodopaminergic phenotype with increased rotatory behavior and decreased TH expression after chronic intranigral delivery of BDNF (Lapchak et al., 1993).

Another important question is that, currently, there is no preclinical animal model of PD that fully demonstrates all of the features of the human disease and this justifies the variability of NTFs results. The neuroprotective and restorative capacity demonstrated by GDNF in neurotoxin-based models is flanked by the failure of GDNF application in α-syn rodent models. Intriguingly, GDNF had no effect on dopaminergic neuron survival and motor symptoms both in a wild-type and A30P mutant α-syn overexpression model of PD (Lo Bianco et al., 2004; Decressac et al., 2011). This failure has been attributed to the decreased level of transcription factor Nurr1, upstream regulator of GDNF receptor Ret whose downregulation block the response to this neurptrophic factor (Decressac et al., 2012). However, the direct correlation between accumulation of α-syn and Ret/Nurr1 downregulation has not yet been totally confirmed (Su et al., 2017).

## The Hard Transition From Pre-Clinical Studies to Human Trials

Previous chapters have highlighted that multiple lines of evidence, derived from preclinical investigations, indicated anomalous expression of various CNS NTFs in neurodegenerative disease involving basal ganglia (Howells et al., 2000; Chauhan et al., 2001).

Theoretically, NFTs may take part to PD pathogenesis. On one hand, it has been hypothesized that the α-syn accumulation may influence the expression of GDNF and BDNF inducing the down-regulation of BDNF transcription and worsening of BDNF trafficking in neurons (Kawamoto et al., 1999, 2000; Yuan et al., 2010; Chu et al., 2012; Pramanik et al., 2017). On the other hand, and aside from the specific interplay between α-syn accumulation and endogenous NTFs, CDNF, and MANF are localized mainly to the lumen of ER and, hence, might play a role in ER stress, *via* the UPR signaling pathways, in neurodegenerative diseases. Both routes (α-syn and mitochondrial energy failure) indicate NFTs as important putative target for therapeutic modulation.

More specifically, since the late 1990s, different post-mortem studies demonstrated the reduction of BDNF protein in SNpc and striatal nuclei (caudate and putamen) of patients with PD (Mogi et al., 1999; Parain et al., 1999), as more recently confirmed by the results in the work of Nagatsu and Sawada (2007). Moreover, *in situ* hybridization data documented the significant reduction of BDNF mRNA in SNpc surviving dopaminergic neurons of patients with PD than their healthy subject counterparts, which express high BDNF mRNA levels (Howells et al., 2000).

The connection between decreased levels of BDNF and PD development has been further outlined *in vivo* by a (123) I-PE2I single-photon emission computer tomography study showing a positive correlation between serum BDNF levels and striatal DAT availability in patients with PD (Ziebell et al., 2012). In addition, GDNF was linked to the dopaminergic system efficiency in PD development. In fact, both striatal and nigral GDNF expression may be markedly decreased in PD. Chauhan et al. (2001) reported a significant reduction of GDNF in the SNpc of patients with PD, which was up to eight times greater than the reduction of other NTFs, thus suggesting that GDNF may be considered as the most vulnerable and earliest NTF to decrease in SNpc neurons that survived to neurodegeneration. Conflicting reports are also available; a lack of difference between healthy subjects and patients with PD, for instance, was documented in a post-mortem study (Mogi et al., 2001).

The translation of those evidence into clinical facts has been hampered by huge limitations. As pointed by Chmielarz and Saarma (2020) “Neurotrophic factor (NTF)–based therapies for PD hold great promise, yet they have so far failed to enter the clinic.” Technical limitations, such as incomplete delivery protocols, and difficulties in selection of the optimal NTF regimen might explain unsatisfactory results with NTFs in PD clinical trials. Here, we summarize some results and pitfalls.

### A Brief Recap on Recent Human Trials Follows

Clinical trials with GDNF date back to over two decades ago. Previously, experience with GDNF did provide inconclusive or, somehow, disappointing results. In particular, the intraventricular GDNF administration (monthly, for 8 months) did not reach any significant benefit, and side effects prevailed (Nutt et al., 2003). On the contrary, the direct delivery (intra-caudate, reminiscent of preclinical studies) appeared to either induce some modest clinical amelioration or PET-confirmed increased functioning of DA neurons (Gill et al., 2003; Love et al., 2005; Slevin et al., 2005, 2007). However, the latter were small open-label studies and the subsequent placebo-controlled one raised more uncertainties (including the development of neutralizing antibodies to GDNF, see Lang et al., 2006; Patel et al., 2013).

Recently, a randomized, double-blind, placebo-controlled trial focused on the intermittent intraputaminal GDNF administration in patients with motor fluctuating PD was conceived (Whone A. et al., 2019; Whone A. L. et al., 2019). This study reached intriguing but still inconclusive results, suggesting some large improvement in OFF state in a sub-cohort of patients with PD. Actually, an open-label phase 1 single-center trial is ongoing (NCT01621581), with safety and tolerability ambitions. It utilizes an adeno-associated virus serotype 2 vector (AAV2) containing human GDNF complementary DNA. In this study, bilateral catheters are placed surgically through the skull into the brain and the vector being delivered by convection-enhanced delivery (CED) to both putamina (450 microliters per hemisphere) of 24 patients with advanced PD. AAV2-GDNF vector represents, indeed, a rather ambitious effort, supposed to run for 5 years in patients with advanced PD, who are currently candidates to surgical DBS therapy; yet, it is not recruiting at the moment, as far as we know. Hopefully, results will be by NINDS along 2022.

### Neurturin and Cerebral Dopamine Neurotrophic Factor

Among the different NTFs tested in clinical trials for PD, three studies using the NRTN as gene therapy stand out. The first study, an open-label trial with intraputamental infusion of CERE-120 (adeno-associated virus serotype 2-neurturin), although it has not shown changing in PET markers, reported patients’ motor improvement in unified Parkinson’s disease rating scale (UPDRS) (Marks et al., 2008). However, these encouraging clinical results were not duplicated in the two subsequent double-blinded studies, where AAV2-NRTN was delivered into putamen (Marks et al., 2010) or simultaneously into both putamen and to SN (Bartus et al., 2013). Nonetheless, NRTN studies, which stratified patients by time from disease diagnosis, indicated some benefits related to in the earliest stage of PD (Marks et al., 2010; Olanow et al., 2015).

As for the studies on CDNF therapy for PD, a double-blind clinical phase I/II trial was started in autumn 2017 (Huttunen and Saarma, 2019). Of note, “CDNF is delivered by a convection-enhanced delivery system once a month for 6 months, which will be followed by an open-label extension period where all patients will be given CDNF.” Results were expected in autumn 2020, but, to our knowledge, they are still missing. However, preliminary data indicate CDNF as a safe and hopeful tool to improve biological activity in PD with possible disease-modifying potentials.

An interesting lesson arises from the abovementioned AAV-NRTN injection studies, as far as post- mortem investigations became possible in two patients, who had received either injections to both putamen and SN or to putamen only, 8 or 10 years after the virus injection (Chu et al., 2020). In these patients, the lack of significant clinical benefit was related to the fact that NRTN expression was limited to 3–12% in the putamen and 9–40% in SN. However, this study proved a large increase of dopaminergic innervation in those putaminal and SN areas where NRTN was express, suggesting a long-term benefits of NRTN in PD. This datum highlights “the capability of NRTN to protect and restore the function of dopamine neurons over a span of almost a decade.” Nevertheless, “saving dopamine neurons in the SN have not improved innervation of the putamen,” as if the already occurred axonal degeneration or perturbed axonal trafficking did impede efficient rescue.

## Putative Modern Strategies: A Role for Focused Ultrasound?

It is extensively known that Magnetic Resonance–guided Focused Ultrasound (MRgFUS) is a versatile tool for clear-cut clinical indications. These clinical applications utilize the high-intensity focused ultrasound (HIFU) protocols. In terms of action mechanism, HIFU causes significant increase in temperature in a defined focal plane of the ultrasound beam, inducing protein denaturation, DNA breakdown, and eventually thermal coagulative necrosis, the latter representing the desired mechanism of action of FUS. In human tissues, therapeutic thermal ablation is achieved at temperatures above 54–56°C. Advances in skull aberration corrective techniques and phased-array transducers have led to the development of fully non-invasive therapeutic transcranial sonication. Combined with MR guidance for targeting, real-time monitoring, and thermal feedback, FUS can be applied through the intact skull and deliver energy to a few millimeters *anywhere* from the cortex to deep lying structure, at selected sites, to produce discrete therapeutic results.

**FIGURE 1:**
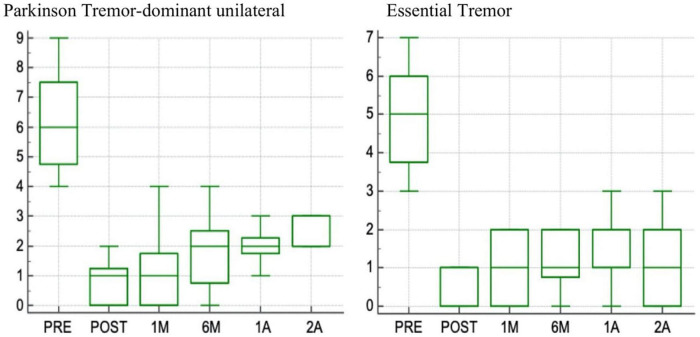
Box plot of changes in scores at the Fahn-Tolosa-Marin (FTM) part A side treated (range 0–9) with 2-years follow up in patients with Parkinson tremor dominant unilateral and in patients with Essential tremor.

We are collaborating with the dedicated team in L’Aquila, which has been applying the procedure, so far, to 67 essential tremor (ET) and 72 patients with PD with significant short-term and long-term reduction in tremor (Figure 1; Bruno et al., 2020a, 2021a).

**FIGURE 2:**
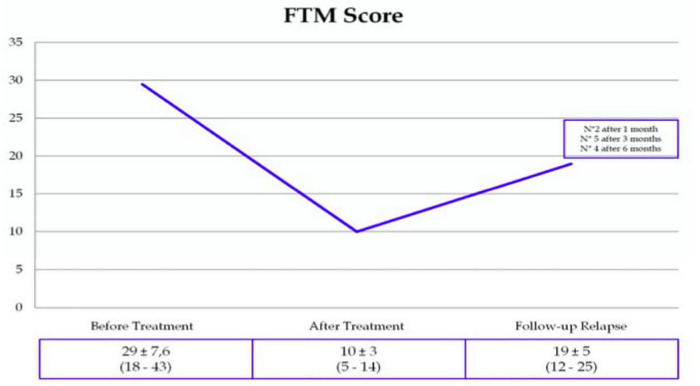
Tremor Recurrence (13%), as estimated in a cohort of MRgFUS-treated ET patients (Sucapane, 1’Aquila).

As several other teams worldwide, they are acquiring experience around potential limitations (i.e., skull density ratio and skull volume in the treatment area, which limit the energy transfer throughout the skull and influence the temperature increase in the lesioning area; the number and maximal average temperature—Tmax-Avg—of sonication during treatment; and the accuracy of targeting, the optimal lesion volume), rare adverse events, and the degree of tremor re-occurrence during follow-up (Bruno et al., 2020b, 2021b; Tommasino et al., 2021; Figure 2).

Aside from the standard indication, which was to neutralize ventral intermediate nucleus and/or the dentatorubral tract and/or pallidothalamic tract to relieve drug-resistant ET, clinical lesioning FUS is currently examined in a variety of other potential indications, from exploring bilateral applications for ET, to revealing possible effectiveness in patients with extremely asymmetric PD, up to the chance, in rather patients with advanced PD, to promoting strategic micro-lesions of the GPi to contain dyskinesias that survived both pharmacological optimization and traditional DBS in STN.

Central to our commentary is the following question: well above the HIFU approach, might MRgFUS provide a tool capable to overcome the limitations so far experienced in terms of delivery of NFTs to CNS?

One of the pitfalls experienced so far, and briefly estimated in the previous chapter, concerns the poor penetration of the administered molecules. The majority of NTFs do not easily cross the BBB (i.e., consider the large size of the GDNF and NTFs homodimer structure). Besides, although different compounds are theoretically able to cross the BBB, the current routes of administration failed to achieve a consistent increase of neuronal BDNF expression or oligodendroglial GDNF in humans, thus hampering the translatable of experimental results into putative disease-modifying therapeutics. Experimental approaches included, i.e., GDNF encapsulated in microspheres composed of biodegradable polymer materials (Garbayo et al., 2009, 2011), DNA nanoparticle gene transfer to achieve long term GDNF expression (Fletcher et al., 2011), encapsulated fibroblasts transfected to produce GDNF and confer behavioral improvements in the 6-OHDA rat model of Parkinson’s disease (Grandoso et al., 2007).

Actually, the *chronic* reduced integrity of BBB might play a detrimental role in neurodegenerative disorders. Previous works from our department indeed emphasized to what extent an altered BBB may correlate with cognitive impairment in patients with advanced PD (Liguori et al., 2017). The issue, here, is instead dealing with what already acclaimed by experimental studies, showing consistently that the temporary and reversible opening through LIFU in conjunction with intravenously administered preformed microbubbles may be strategic.

Among the applications of the MRgFUS, the ability of this technique to transiently increase the BBB permeability, through the temporary and focalized (localized) opening of BBB tight junctions, should be emphasized. Further, the transient and reversible approach with MRgFUS, different from other pioneering methods proposed to improve CNS-drug delivery, such as receptor modifying nanoparticles, intranasal (IN) injections, and chemo-agent wafers (Gabathuler, 2010; Lochhead and Thorne, 2012; Bregy et al., 2013; Torres-Ortega et al., 2019) should not imply (or render negligible) the risk of iatrogenic injury or irreversibly toxicity. There are several experimental studies of opening the BBB on patients with brain tumors that show the safety and feasibility of the procedure (Lee et al., 2019; Chen K.T. et al., 2021). In recent years, FUS has been applied for BBB opening for therapeutic purposes also in AD and other neurological diseases.

Actually, mechanisms underlying LIFU are quite complex, given that modulatory functions on neurons and glial cells, either neuroexcitatory (i.e., membrane depolarization) or neuroinhibitory depend upon on the ultrasound parameters, whose evaluation is well above the scopes of this manuscript. However, for our purposes (delivery of NTFs), it is, of note, that pulsed LIFU *at controlled energy* is important, since being capable to produce stable oscillation of microbubbles, which transiently opens the BBB by separating the endothelial tight junction, enhancing the localized delivery of therapeutic agents including antibodies, growth factors, nanoparticles, nucleic acid, viral vectors, and cells to the brain (Sheikov et al., 2004; Fishman and Frenkel, 2017; Lee et al., 2019). On the other hand, high energy might induce inertial cavitation and bubbles collapsing. Several studies focus on the enhancement of the acoustic pressure below a certain threshold to render the delivery procedure safe. A recent review clarifies the modalities of application at both end of the acoustic spectrum (Conti et al., 2021). In humans, MR guidance is the routine, although recent experience on two non-human primates have been designing “the feasibility of BBB opening, through a portable, robotic-assisted clinical system with a therapeutic transducer able to promote real-time cavitation monitoring (hence, provided the availability of neuronavigation system, eliminating the constant need of MRI)” (Pouliopoulos et al., 2020).

First applications, quite surprisingly, date back to the early 2000s when Hynynen et al. (2001) applied LIFU, instead of HIFU, in conjunction with intravenously administered preformed microbubbles for BBB opening.

At present, the big question here is to what extent FUS, effectively experienced in disease models, can definitively help the implanting of microparticles or transfected cells in the human brain. A brilliant review (Lapin et al., 2020) was recently confirming that “a method that overcomes those limitations is FUS in the presence of systemically circulating microbubbles” (Sheikov et al., 2004; Meijering et al., 2009; Chopra et al., 2010). Indeed, FUS has been conceived as a therapy-delivering approach for malignancies, neurodegenerative diseases, and movement disorders (Lin et al., 2015, 2016; Szablowski et al., 2018; Chen K.T. et al., 2019). Albeit the transition from laboratory to daily clinical practice remains hard, pioneering investigations are emerging. In Alzheimer’s rodent’s models, it was shown that FUS-induced BBB permeability represents a critical requirement to deliver a significant amount of intravenous immunoglobulin (489 ng/mg) to the targeted hippocampus of TgCRND8 mice (Dubey et al., 2020); see also the MRgFUS-mediated neurogenesis and improved cognition, promoted by the allowed delivery of a TrKA agonist in the TgCRND8 model of AD (Xhima et al., 2021). In humans, an ongoing protocol is fascinating. Rezai et al. (2020) lately presented the “initial clinical trial results evaluating the safety, feasibility, and reversibility of BBB opening with FUS treatment of the hippocampus and entorhinal cortex (EC) in patients with early AD.” Post-FUS in contrast to MRI revealed immediate and sizable hippocampal parenchymal enhancement indicating BBB opening, followed by BBB closure within 24 h, hence favoring FUS as a safe, non-invasive, transient, reproducible, and focal mediator of BBB opening in the hippocampus/EC in humans.”

Next paragraph will focus on the current evidence for the efficacy of FUS approach in experimental PD models (as premise for translational applications).

## Focused Ultrasound-Assisted Approach for Delivering NTFs in Parkinson’s Disease Models

As previously reported, FUS in conjunction with intravenously injected microbubbles induce a safe, non-invasive, and reversible enhancement of BBB permeability. Since 2012, the key parameters in determining drug delivery across the BBB (namely, time window and molecular size) were estimated with mathematical models (Marty et al., 2012). The therapeutic application of BBB opening by FUS for the delivery of NTFs has proved, indeed, efficacious in different experimental PD models (Karakatsani et al., 2019a).

The potential of FUS method to deliver neuroprotective agents had received a seminal support by Samiotaki et al. (2015), whose experiments showed an efficient NRTN bioavailability in both the SN and CP through the optimization of acoustic parameters. Moreover, the authors demonstrated the activation of NRTN signaling pathway, i.e., intracytoplasmic increase of Erk1/2 phosphorilation and the downstream transcription factor cAMP response element-binding protein (CREB), playing a well-known role inside the nigrostriatal circuitry.

Another report that addressed BBB opening and neurotrophin delivery in a PD model combined FUS and transvascular non-viral gene delivery system (microbubbles conjugated with GDNF-plasmid) (Fan et al., 2016). This strategy showed a meaningful recovery of DA concentration and restoration of behavioral function in a standard 6-OHDA–lesioned PD model (Fan et al., 2016). Interestingly, Karakatsani et al. (2019b) investigated a “more discrete” PD model in mice, obtained following subacute MPTP injections (leading to an apoptotic-like, partially reversible degeneration, reminiscent of an “early” PD). In this model, it was shown that only the combination of FUS and intravenous NFT delivery (NRTN) increased unilateral TH staining in SN; further, only repeated FUS/NRTN exposures were able to recover the TH fibers density in caudate putamen (CPu), hence affecting DA release, as quantified by HPLC-determined levels of major metabolites, homovanillic acid (HVA) and 3,4-dihydroxyphenylacetic acid (DOPAC), in midbrain and striatum (Karakatsani et al., 2019b).

A clever synergistic use of FUS and gene-carrying liposome to perform non-invasive GDNF delivery was proposed in two different PD models, respectively, MPTP-treated mice (Lin et al., 2016) and 6-OHDA–lesioned rats (Yue et al., 2018a,b). In this case, MRI-guided singleFUS treatment of 6-OHDAlesioned striatum, combined with brain-penetrating polymeric nanoparticles, induced relevant GDNF level increase in the target region, lasting up to 10 weeks after treatment, and a prolonged improvement in locomotor function (Mead et al., 2017).

Last but not least, the IN route has opened new avenues: the first demonstration dates back to 2016, when fluorescence imaging of brain slices found that IN administration of BDNF followed by FUS sonication achieved a significant enhancement of BDNF distribution in FUS-treated CPu compared with the corresponding controls (Chen et al., 2016). These preliminary data were extensively applied in Ji et al. (2019) to an early stage PD mouse model in which the up-regulation in TH expression and the corresponding behavioral changes indicated that applied FUS-mediated-BBB opening improved the delivery of IN BDNF into the target regions.

So far, a rapid translational road is still uncertain. Whether analogous approach would be efficacious for patients with advanced PD is matter of present research (Foffani et al., 2019; Fishman and Fischell, 2021). It will be critical to minimize possible adverse events and concerns, as brilliantly summarized by Todd et al. (2020).

## Conclusion

- Bioavailability of NTFs in the target tissue remains a major challenge for NTF-based therapies; yet, contradictory results should not discourage our efforts.
- The difficulty to solve NTFs delivery (Marty et al., 2012; Conti et al., 2019) was partially overcome by revisiting FUS approach through multimodal protocols.
- MRgFUS, through dedicated approach designed to transiently modify the BBB permeability, may indeed be considered a safe method of relatively simple implementation, and, likely, effective when compared to the surgical procedures, which administered NTFs in specific intracerebral target nuclei by stereotactic delivery, either directly (Patel et al., 2005; Lang et al., 2006) or *via* viral vectors (Bartus et al., 2013; Olanow et al., 2015). Consistent experience with FUS-facilitated delivery of neurotrophic agents, either assisted by MR guidance or not, accumulated in experimental PD models and has paved the road for ongoing and up-coming clinical trials.
- However, in many respects, combined strategies should not be excluded. Functional neurosurgery is undergoing a fascinating renovation (Stefani et al., 2017; Priori et al., 2021), and multi-disciplinary teams are welcome. In this content, some experimental studies utilize synergistic approach: a Finnish group, for instance (Huotarinen et al., 2018), detected that “CDNF delivery improved the effect of acute STN DBS on front limb use asymmetry at 2 and 3 weeks after CDNF injection.”

The ambition of promoting neuro-rescue, in PD and other movement disorders, so far unmet need, will require an abundant dose of experimental courage and clinical wisdom.

## Author Contributions

All authors listed have made a substantial, direct, and intellectual contribution to the work, and approved it for publication.

## Conflict of Interest

The authors declare that the research was conducted in the absence of any commercial or financial relationships that could be construed as a potential conflict of interest. The reviewer AC declared a shared affiliation, with several of the authors AS, MP, LS, RC, MC, and SC to the handling editor at the time of the review.

## Publisher’s Note

All claims expressed in this article are solely those of the authors and do not necessarily represent those of their affiliated organizations, or those of the publisher, the editors and the reviewers. Any product that may be evaluated in this article, or claim that may be made by its manufacturer, is not guaranteed or endorsed by the publisher.
